# Oxygen mass transfer in a stirred tank bioreactor using different impeller configurations for environmental purposes

**DOI:** 10.1186/1735-2746-10-6

**Published:** 2013-01-07

**Authors:** Ali Karimi, Farideh Golbabaei, Momammad Reza Mehrnia, Masoud Neghab, Kazem Mohammad, Ahmad Nikpey, Mohammad Reza Pourmand

**Affiliations:** 1 Department of Occupational Health, School of Public Health and Nutrition, Shiraz University of Medical Sciences, Shiraz, Iran; 2 Department of Occupational Health, School of Public Health, Tehran University of Medical Sciences, Tehran, Iran; 3 Biotechnology Group, School of Chemical Engineering, University College of Engineering, University of Tehran, Tehran, Iran; 4 Department of Epidemiology and Biostatistics, School of Public Health, Tehran University of Medical Sciences, Tehran, Iran; 5 Department of Occupational Health, School of Public Health, Qazvin University of Medical Sciences, Qazvin, Iran; 6 Department of Pathobiology, School of Public Health, Tehran University of Medical Sciences, Tehran, Iran

**Keywords:** Stirred tank bioreactor, Oxygen mass transfer coefficients, Impellers

## Abstract

In this study, a miniature stirred tank bioreactor was designed for treatment of waste gas containing benzene, toluene and xylene. Oxygen mass transfer characteristics for various twin and single-impeller systems were investigated for 6 configurations in a vessel with 10 cm of inner diameter and working volume of 1.77L. Three types of impellers, namely, Rushton turbine, Pitched 4blades and Pitched 2blades impellers with downward pumping have been used. Deionized water was used as a liquid phase. With respect to other independent variables such as agitation speed, aeration rate, type of sparger, number of impellers, the relative performance of these impellers was assessed by comparing the values of (K_L_a) as a key parameter. Based on the experimental data, empirical correlations as a function of the operational conditions have been proposed, to study the oxygen transfer rates from air bubbles generated in the bioreactor. It was shown that twin Rushton turbine configuration demonstrates superior performance (23% to 77% enhancement in K_L_a) compared with other impeller compositions and that sparger type has negligible effect on oxygen mass transfer rate. Agitation speeds of 400 to 800 rpm were the most efficient speeds for oxygen mass transfer in the stirred bioreactor.

## Introduction

Benzene, toluene and xylene (BTX) as hazardous volatile organic materials from various emission sources such as oil and gas refineries, petrochemical industries, shoe-making manufactures, printing and paint manufacturing industries, are considered as great threat to the public health and the environment. Biotreatment with the advantages of high efficiency, low-cost, and non-secondary pollution is suitable to purify waste gas in low concentrations [1-4].

In many biochemical processes the oxygen supply to the broths is not enough to meet the demand of the microorganisms. Oxygen transfer is often the limiting factor in the aerobic bioprocess due to the low solubility of oxygen in the medium; so aeration is a critical factor in industrial aerobic fermentations [5-9].

In stirred tank bioreactors the oxygen mass transfer is a function of many variables, such as the physical properties of the liquid (viscosity, surface tension, etc.), the geometry of the vessel and stirrer, the type of sparger and the operational conditions. Unfortunately, the available information in the literature about the effect of these variables on the mass transfer is sometimes confusing [8,10].

Stirred tank bioreactors provide high values of mass and heat transfer rates and excellent mixing. In these systems, a high number of variables affect the mass transfer and mixing, but the most important among them are stirrer speed, type and number of stirrers and gas flow rate used [6,11]. The most important role is played by the impeller, which accomplishes three major tasks, solids suspension, mixing and dissolution of the required atmospheric oxygen into the aqueous phase, and maximizing the interfacial area between the gaseous and aqueous phases [8,12]. The most studied impellers have been the standard Rushton turbines, different pitched blade turbines and various propellers as well as combinations of two or three of them to optimize the power consumption [13-15].

Fujasova [9] studied the mass transfer rate of seven types of impellers in 29 triple configurations. They found that Rushton turbine impeller in triple configuration and combination of Rushton turbine with Pitched blade are the most efficient impeller combinations for the mass transfer performance in the triple-impeller vessel. Tomoa Moucha *et al*. [16] reported that the conclusions about the influence of impeller configuration on the mass transfer efficiency are ambiguous. They reported that this is partially caused by the improper methods used for volumetric oxygen transfer coefficient (K_L_a) data evaluation and usually all phenomena were not taken into account, which affects the results.

Extensive investigations on K_L_a have been conducted by previous researchers, especially for reactors using conventional impellers. Several studies are also available in the literature that have investigated different aspects of oxygen transport in different works [17,18].

To optimize the impeller design for effective gas dispersion, it is essential to understand the mechanism of better oxygen mass transfer performance, so the present work on the bioreactor design was directed towards the study of oxygen transfer and its availability in the bioreactor. In this study, design and construction of a laboratory scale stirred tank bioreactor was followed by measurements of K_L_a in the aerated bioreactor in order to identify the optimal operational conditions of the oxygen mass transfer from gas into the aqueous phase. The independent variables were: type of impellers, number of impellers, aeration rate, agitation speed and types of sparger.

## Materials and methods

### Bioreactor configuration

**Figure 1:**
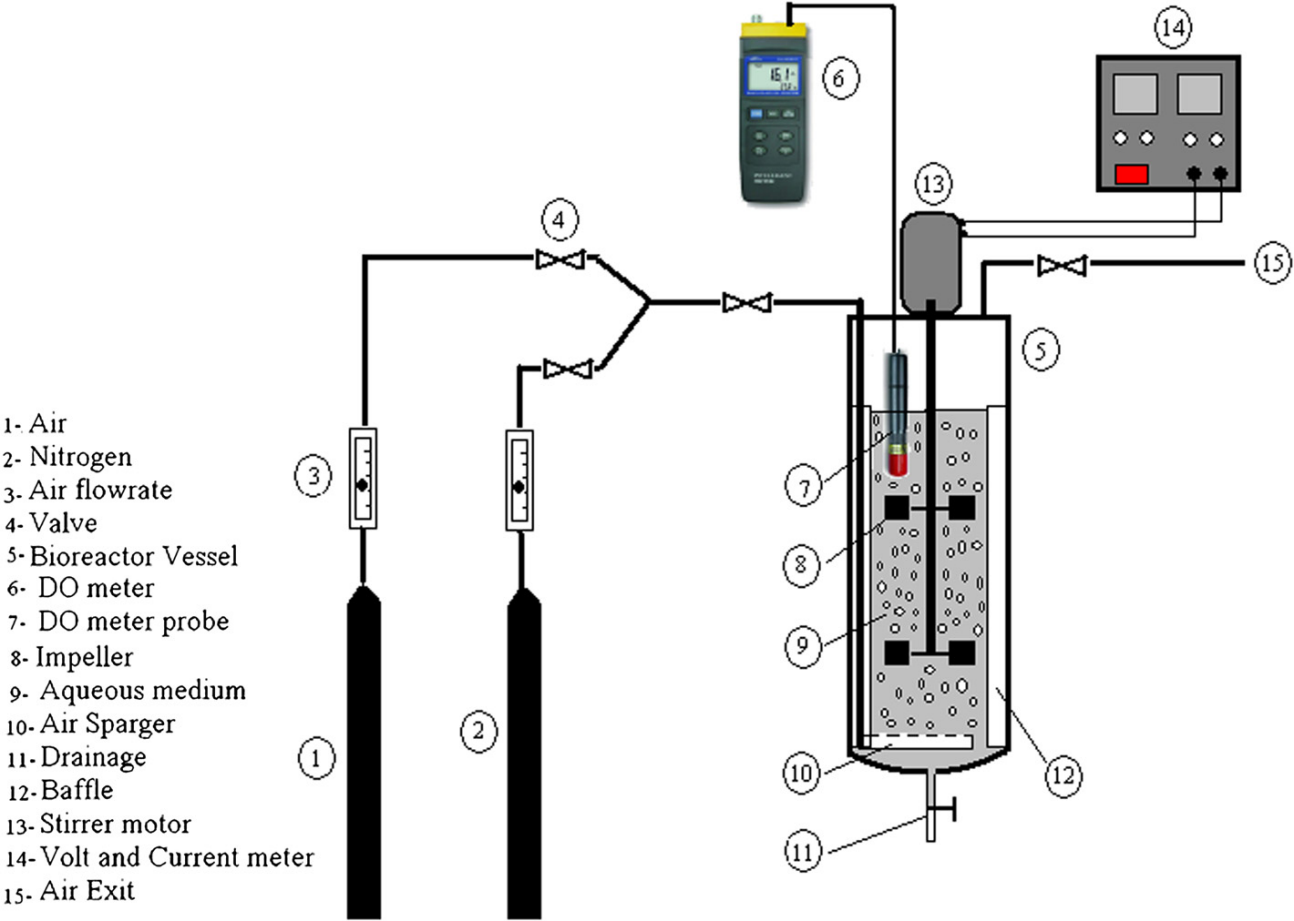
Schematic view of stirred tank bioreactor used in this study.

**Figure 2:**
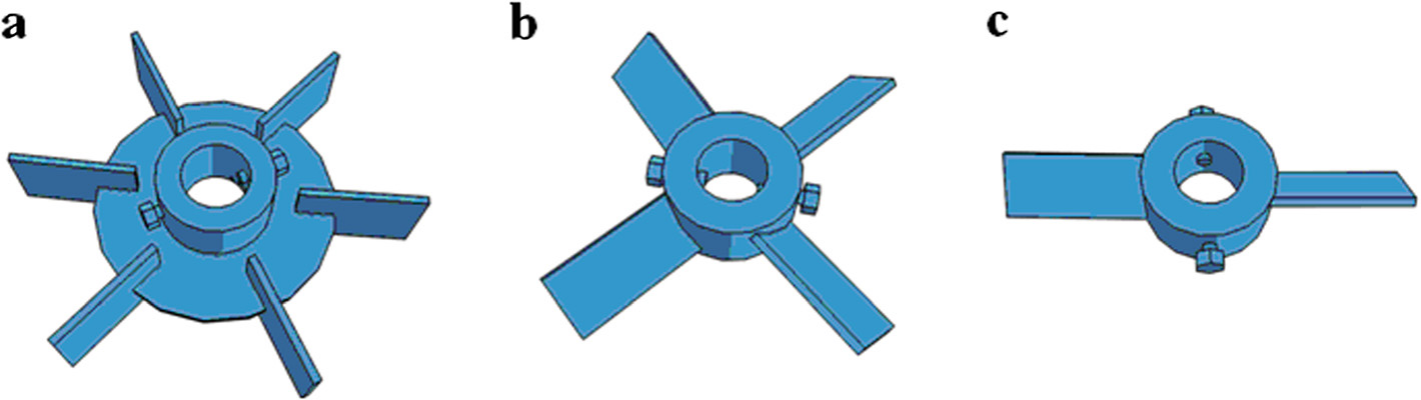
**Schematic view of the three types of impellers**, **a: Standard Rushton turbine with vertical Blades(RT), b: Pitched 4blade (P4B)**, **c: Pitched 2blade (P2B).**

Experiments were performed in a cylindrical vessel. The semi-circle bottom of the cylindrical vessel was made of transparent glass with an internal diameter of T_i_=10 cm and fitted with four vertical wall baffles symmetrically. The experiments were performed in semi-batch conditions at room temperature and atmospheric pressure. The liquid phase was deionized water (28± 0.5°C). Filtered air was fed to the system through the sparger located 5cm (T_i_/2) below the lower impeller. The experimental conditions have been selected in order to generate normal flow patterns inside the tank. Figure 1 and Table 1 give the schematic and dimensions of stirred tank bioreactor designed, constructed and utilized in this study. The schematic view and details of the three types of impellers are shown in Figure 2 and Table 2, respectively. Since mass transfer depends on the bubble size, three perforated tubes were also used as sparging devices (Table 3).

**Table 1:** Characteristics of the stirred tank bioreactor

**Descriptions**	**Unit**	**Value**
Body of vessel (material)	-	Glass (semi-circle bottom)
Internal diameter of vessel (T_i_)	cm	10
external diameter of vessel (T_e_)	cm	11
Vessel height(H)	cm	30
Vessel aspect ratio (H:T_i_)	-	3 : 1
Bottom impeller clearance	cm	5 (T_i_/2)
Distance between impellers, (h_1_)	cm	10 (T_i_)
Height of stirrer motor shaft	cm	25
Working volume (V_L_)	liter	1.77 (75%)
Number of baffles	-	4
Baffles width (w_b_)	cm	1 (T/10)
Impellers type	-	RT , P4B , P2B
Number of impellers	-	One and two
Sparger types	-	Orifice and nozzle

**Table 2:** Details of impellers used

	**Rushton Turbine** (**RT**)	**Pitched 4**-**blades** (**P4B**)	**Pitched 2**-**blades** (**P2B**)
Impeller diameter, cm (D_i_)	5 (T_i_/2)	5 (T_i_/2)	5 (T_i_/2)
Impeller blade width, cm (w_i_)	1	0.6	0.6
Impeller blade length, cm (l_i_)	1.5	1.75	1.75
Number of blades	6	4	2
Ratio of impeller diameter to tank diameter (D_i_/T_i_)	1: 2	1: 2	1: 2
Flow direction	Radial	Axial	Axial
Inclination (degree °)	0	45	45

**Table 3:** Details of spargers used

	**Sparger 1**	**Sparger 2**	**Sparger 3**
Type	Nuzzle	Orifice	Orifice
Number of holes	1	4	9
Hole's diameter, mm	6	3	2
Total open area, mm^2^	28.3	28.3	28.3

### Impellers

Three types of impellers, namely Rushton turbine (RT), Pitched 4blade (P4B) and Pitched 2blade (P2B) impellers (blade angles 45°) with downward pumping (Figure 2) were tested with 1.77 L total working volume of deionized water in order to evaluate their effect on the oxygen mass transfer rate from gas to liquid phase in the bioreactor. Aeration rates of 1, 2, 3, 4 and 5 L/min were tested in eleven agitation speeds (0, 100, 200, 300, 400, 500, 600, 700, 800, 900 and 1000 rpm). Higher gas flow rates overwhelmed the bioreactor vessel.

### Oxygen mass transfer coefficient

Gas-liquid contact is a matter of decisive importance for describing systems involving biological processes. Mass transfer between phases may often become the limiting step of the overall process rate. In such cases, the volumetric oxygen transfer coefficient, K_L_a*,* must be known in order to carry out the design and scale up of bioreactors [10]. The mass balance for the dissolved oxygen in the well-mixed liquid phase, in the absence of biomass or with non-respiring cells when biochemical reactions do not take place, can be established as follows [6]:

(1) $$\frac{\mathrm{dC}}{\mathrm{dt}}=K_{L}a.\left(C^{*}-C\right)$$

Where C is the oxygen concentration, dC/dt is the accumulation oxygen rate in the liquid phase, C* is the equilibrium dissolved oxygen concentration. Some measuring methods are based on Eq. (1) and different techniques for measuring the dissolved oxygen concentration can be used.

The volumetric coefficient of oxygen transfer from gas to aqueous phase was determined by the dynamic gassing-out method [7,19]. This technique is interesting for studying the influence of operational conditions on the volumetric mass transfer coefficient and is widely employed in the literature [5,6,8,20-22].

The dissolved oxygen concentration was monitored with an oxygen electrode, Lutron oxygen meter moddle YK- 2001 DO, fitted with a teflon membrane and with and electrolytic solution of Na_3_PO_4_ in the cell.

### Gas dissipating device (sparger)

Three types of gas spargers with different dimensions were tested in the stirred bioreactor. Details of used spargers are presented in Table 3. This part of study aimed to answer the question if the sparger designs have any influence on mass transfer rate of oxygen.

### Correlations for oxygen mass transfer coefficients

Predictions of the rate of absorption of a gaseous material in a stirred tank are usually based on correlations of overall volumetric mass transfer coefficient (K_L_a) with mechanical agitation power per unit volume (P/V_L_) and gas sparging rate expressed as the superficial velocity (V_g_) [19]. The power input per unit volume (P/V_L_) and superficial gas velocity V_g_ are major factors in these K_L_a correlations.

There are a lot of proposed equations for the volumetric mass transfer coefficient as a function of different variables in previous studies [8,23]. The following equation is frequently found in the literature [19,24,25]:

(2) $$K_{L}a=\alpha {\left(\frac{P_{g}}{V_{L}}\right)}^{\beta }{\left(V_{g}\right)}^{c}$$

Where:P_g_: the mechanical agitation power in gas liquid dispersion (W); V_L_: liquid volume (m^3^); V_g_: gas superficial velocity (m/s); α: constant; β and c: exponents.

The P_g_ was measured by electrical measurement method, using a circuit control that monitored the electrical current (A) and voltage (V) of the DC stirrer motor mounted on the bioreactor. V_L_ as the working volume of the bioreactor in this study was 0.177 m^3^ and V_g_ was calculated via dividing the gas flow rate (m^3^/s) by internal tank area (m^2^).

### Correlations

Some empirical correlations for the oxygen transfer rate in a the bioreactor with three types of single and twin-impellers are developed and K_L_a values obtained from the experimental data were plotted against the operating variables and mathematical correlations which describe the influence of the studied parameters on the K_L_a have been established in order to predict biodegradation performances from view points of oxygen mass transfer, when using models that account for the effect of dissolved oxygen. These correlations ware developed using Datafit 9 software.

## Results

To evaluate the optimal conditions of the bioreactor, the effect of oxygen mass transfer rate in several operational conditions including different types of impellers and spargers in various agitation and aeration rates were tested.

**Figure 3:**
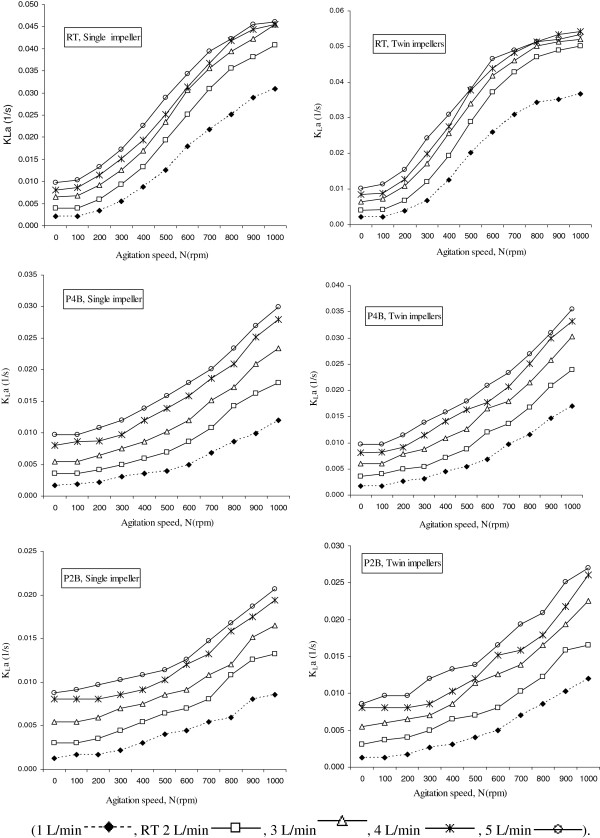
**K**_**L**_**a as a function of impeller speeds** (**rpm**) **with flowrates of 1 to 5 L**/**min using three types of impellers** (**RT**, **P4B and P2B**) **in 6 different single and twin configurations.**

**Figure 4:**
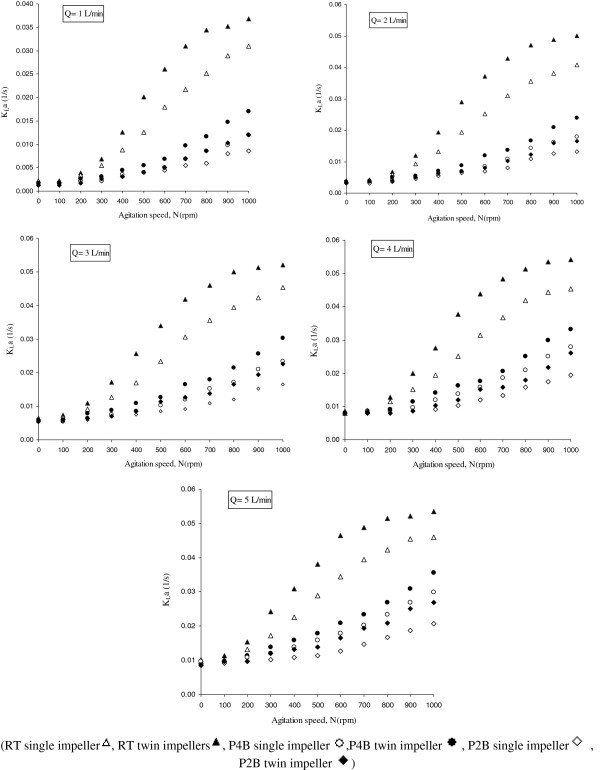
**Performance comparisons of three types of impellers** (**RT**, **P4B and P2B**) **in 6 different single and twin configurations from viewpoint of oxygen mass transfer in different flowrates** (**1 to 5 L**/**min**)**.**

Figures 3 &4 illustrate the results obtained showing that the impeller which produces higher K_L_a values is the type RT for all the aeration rates and agitation speeds studied.

The effect of the number of blades on the enhancement of the K_L_a has been studied using two impellers, with 2 and 4 pitched blades geometrically identical (Figure 2).

Table 4 gathers the values of K_L_a in the absence of agitation, in order to compare the effect of the impeller on the mass transfer rate.

**Table 4:** Mass transfer in the absence of agitation

**Q** (**L**/**min**)	**K**_**L**_**a** (**1**/**s**)
1	1.4 ×10^-3^
2	3.4 ×10^-3^
3	5.7 ×10^-3^
4	7.7 ×10^-3^
5	9.3 ×10^-3^

**Figure 5:**
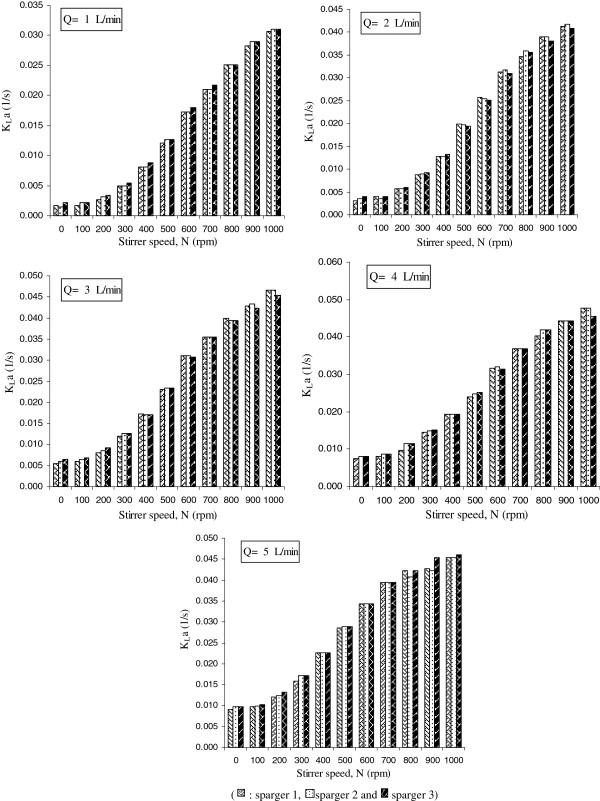
**Effect of three types of spargers** (**1**, **2 and 3**) **on the K**_**L**_**a values in various agitation speeds** (**N**) **and different flowrates** (**Q**)**.**

The results in Figure 5 illustrate the effect of sparger type on K_L_a in different flowrates in the bioreactor.

**Figure 6:**
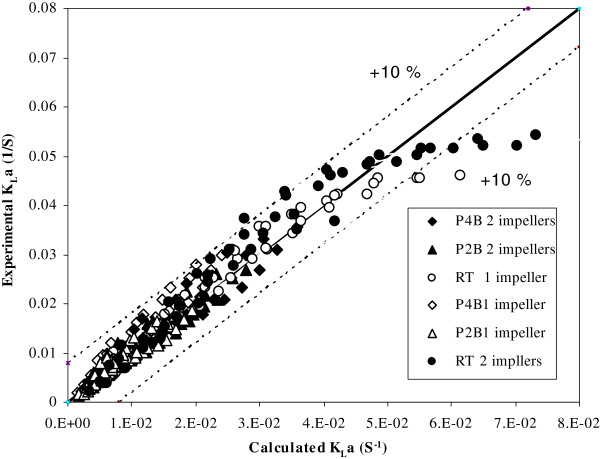
**Correlation between the experimental and calculated values of K**_**L**_**a for single and twin impellers of RT**, **P4B and P2B in six configurations**, **agitation rates from 100 to 1000 rpm and aeration rates from 1 to 5 L**/**min.**

Figure 6 plots the experimental K_L_a for three types of the impellers, the RT, P4B and P2B with twin and single configurations, versus power input including k_L_a calculated using Eq. (2) for different gas flow rates (1 to 5 L/min) and agitation speeds (100 to 1000 rpm).

## Discussion

In Figure 3, it can be seen that for RT impeller type the K_L_a increment trends in different aeration rates(1-5 L/min) begins from a plateau condition in agitation speeds of 0-100 rpm and continues with a noticeable enhancement that can be seen from 200 to 800 rpm; then this trend approaches a plateau in 900 and 1000 rpm. It seems that the most efficient mixing is obtained in agitation speeds more than 300 rpm and lower than 800 rpm. On the other hand for P4B impellers the K_L_a increment trends in different aeration rates(1-5 L/min), begins from a plateau condition in agitation speeds of 0-200 rpm and continues with inefficient enhancement that starts from 300 rpm and does not reach to plateau in high agitation speeds. The K_L_a increment trend, using P2B impeller has shown similar pattern compared to P4B impeller with this difference that K_L_a increment starts at agitation speed of 400 rpm.

The impeller type RT represented an average enhancement on the K_L_a values of 50% to 69 % and 60 to 77% with respect to impellers P4B and P2B, respectively (in twin impeller configurations). This impeller is more efficient in breaking the air bubbles because it has a higher transversal section area and, consequently, it increases the superficial area of the bubbles, enhancing the oxygen transfer rate. However at lower agitation speeds this difference is not so effective, and at 0 to 200 rpm, the K_L_a values do not present significant differences using different types of impellers studied.

There is only a significant enhancement on K_L_a values for the medium agitation rate (400 to 800) that could be resulted from the higher breakage and residence time of the air bubbles in the bioreactor media. The size of the drops in a mixing vessel is largely dependent on the micro and macro-scale turbulent motions and flow patterns in the vessel because of the mutual relation between the local energy dissipation rates, the residence time of the drops at a certain location in the vessel, and the local breakup or coalescence rates of the drops [5].

As shown in Figure 4, the higher number of blades in impellers has noticeable effect on mixing conditions and volumetric mass transfer of oxygen. The range of K_L_a enhancement showed to be 15% to 27% and 20% to 28% in single and twin configurations, respectively. It seems that there is an increment in the circulation and the contribution of the surface aeration, and the higher number of blades will also increase the break up of bubbles and so the K_L_a would increase. Also twin impeller configurations have shown higher volumetric oxygen mass transfer to compare with single impeller configurations in all types of impellers (Figure 4).

For low agitation rates (0 to 300 rpm) the turbulence is not enough to trap and hold up the air bubbles and consequently performance of volumetric mass transfer may not increase noticeably. Therefore based on the results obtained, agitation speed of 400 to 800 rpm would be beneficial for all the future bioprocess operations that may lead to a higher productive biomass system.

Figure 4 shows that for most of conditions studied the agitation proved to be more efficient in K_L_a enhancement than the aeration. This behavior is in agreement with the results of Chen *et al*. and Amaral [5,23].

The results in Figure 5 showed that in stirred vessels, design of the sparger and the mechanics of bubble formation are of secondary importance compared with the effects of the impeller. When the sparger is located under the stirrer, it has been shown that sparger type does not significantly affect mass transfer. Also Oosterhuis *et al.* has noted that correlations related to K_L_a in stirred bioreactors do not depend on the sparger or stirrer design [26].

The constants obtained from the correlations of the 6 single and twin impeller configurations are shown in Table 5. The exponent over (P_g_/V) increased with single to twin impeller configurations in all types of impellers, indicating more effective utilization of the power with multiple impeller systems. No significant effect was observed on the exponent over V_g_ for single and twin impeller systems. Also in Figure 6 it can be seen that the experimental K_L_a turned out to lay within the values predicted. Empirical correlations for the volumetric mass transfer coefficient depend on several geometrical parameters, although there is no agreement in the literature about how to take into accounts this influence [6].

**Table 5:** **Calculated constants and exponents of Eq**. **2 for 6 impeller configurations**

**Configuration**	**Mass transfer coefficient correlation**, **exponents of Eq**. **2**			
	**α**	**β**	**c**	**R**^**2**^
RT, single impeller	0.28	0.67	0.58	0.93
RT, twin impeller	0.33	0.68	0.58	0.81
P4B, single impeller	1.22	0.55	0.77	0.93
P4B, twin impeller	0.82	0.60	0.72	0.93
P2B, single impeller	1.16	0.46	0.75	0.90
P2B, twin impeller	1.64	0.55	0.81	0.94

## Conclusions

Evaluation of the experimental data shows that K_L_a values are affected by process variables such as impeller configuration, impeller speed and aeration rate. From the above discussion it is clear that twin impeller showed better results in different types of impellers. As the results have shown, twin Rushton turbine represent an average enhancement on the K_L_a values of 50 to 69% and 60 t0 77% with respect to P4B and P2B impellers respectively. It was found that agitation speeds of 400 to 800 rpm would be beneficial for all the future bioprocess operations that may lead to a higher productive biomass system. Also it was observed that with an increase in the gas flow rates, the K_L_a values increased and higher number of blades in identical impeller types resulted in noticeable enhancement of K_L_a in the stirred tank bioreactor.

## Competing interests

The authors declare that they have no competing interests.

## Authors’ contributions

AK and FG participated in the design of the study. AK, MRM and KM performed the statistical analysis. AK carried out the experimental studies. MN, AN and MRP helped to draft the manuscript. All authors read and approved the final manuscript.
